# Survey of *Mycobacterium* spp. in Eurasian Badgers (*Meles meles*) in Central Italy

**DOI:** 10.3390/ani14020219

**Published:** 2024-01-09

**Authors:** Elga Ersilia Tieri, Lucio Marino, Katiuscia Zilli, Cinzia Pompilii, Giovanni Di Teodoro, Antonio Cocco, Addolorato Ruberto, Michela Toro, Maria Teresa Mastrodomenico, Stefania Salucci, Fabrizio De Massis

**Affiliations:** Istituto Zooprofilattico Sperimentale dell’Abruzzo e del Molise ‘G. Caporale’ (IZS Teramo), Campo Boario, 64100 Teramo, Italy

**Keywords:** badger, DNA typing, Italy, *Mycobacterium avium* subsp. *avium*, *Mycobacterium avium* subsp. *paratuberculosis*

## Abstract

**Simple Summary:**

The Eurasian badger (*Meles meles*) is a wild carnivore well adapted to the Abruzzo and Molise regions. During the 2013–2021 period, badger carcasses found dead or road-killed were systematically subjected to an anatomopathological examination and search for mycobacteria with the aim of ascertaining their presence in this species, given the absence of data in Italy. This study provides evidence of infection with the *Mycobacterium avium* subsp. *avium* (MAA) and *M. avium* subsp. *paratuberculosis* (*MAP*) in a small number of badgers. *M. bovis* does not seem to circulate within badger populations. This study suggests that in Central Italy, *Meles meles* should not play a role in the epidemiology of these mycobacteria under current epidemiological conditions.

**Abstract:**

A survey to determine the presence of *Mycobacterium* spp. in the Abruzzo and Molise regions was conducted by testing samples from 124 badgers found dead or road-killed during the 2013–2021 period. Head lymph nodes were collected from all carcasses, as well as mediastinal lymph nodes from 20 of them, for bacteriological and molecular tests; tissues were inoculated onto a set of solid egg-based Lowenstein–Jensen media and in a liquid culture system (BACTEC) and were analyzed by polymerase chain reactions (PCRs). Organs and lymph nodes from 31 carcasses were collected for histological tests. During post-mortem examinations, macroscopic lesions consistent with a *Mycobacterium tuberculosis* complex (MTBC) and with nontuberculous mycobacteria (NTM) infections were not detected. Mycobacteria were isolated from four animals (3.22%). *M. avium* subsp. *avium* was isolated by head lymph nodes from two badgers (1.61%), *M. avium* subsp. *paratuberculosis* (0.80%) from one, and *Mycobacterium* spp. from another (0.80%). The significance of nontuberculous mycobacteria (NTM) in wildlife hosts in the absence of clinical signs and gross pathology has yet to be assessed. The most critical aspect came from isolates belonging to the *Mycobacterium avium* complex infection in wildlife due to the possible interference with tuberculin skin tests in cattle.

## 1. Introduction

Mycobacteria are classified as tuberculosis mycobacteria capable of triggering tuberculosis in humans and animals, and they belong to the *Mycobacterium tuberculosis* complex (MTBC) group, including *M. bovis*, and in nontuberculous mycobacteria (NTM), which cause diseases other than tuberculosis. In the group of NTM, there are members of the *Mycobacterium avium* complex (MAC), which includes the species *Mycobacterium avium* and its subspecies *M. avium* subsp. *avium* (MAA) and *M. avium* subsp. *Paratuberculosis* (*MAP*) [1,2].

Wildlife is susceptible to the same infectious agents that affect livestock species, and where livestock is managed under extensive farming, cross-transmission may be favored [3].

The Eurasian badger (*Meles meles*) is widely distributed across Europe; however, from several countries, epidemiological information about tuberculosis on this species is lacking, making it difficult to assess the role of these badgers as a wildlife tuberculosis reservoir in Europe [4].

*Meles meles* seems to be involved in the transmission of *M*. *bovis* in cattle in the United Kingdom (UK) and Ireland, where badgers share pastures with cattle, which are extensively farmed [5,6]; in these territories, the tuberculosis prevalence is high in badgers and in cattle, probably due to the behavior, the high abundance, and the social group size with a greater number of individuals. Between 1998 and 2005, the prevalence rates of *M. bovis*, estimated by culture in the UK during the randomized badgers culling trial varied from 1.6% to 37.2%, with an average of 16.6%. In the same period, the badger population density was 3.92 badgers/Km^2^, and on initial proactive culls, the mean estimated social group size was 5.44 (±4.27) [5]. In Ireland, in the period of 2002–2003, the prevalence of tuberculosis estimated by bacteriological examination of a wide range of anatomical site tissues of culled badgers was 43.2% [6]; in the period of 1993–2009, researchers reported a population density of 1.9 badgers/Km^2^ and a social group size between 1.8 to 5.9 [7]. Although studies on badger tuberculosis differ both in the target population (road-killed or culled), in sample size, and in the diagnostic method used (gross lesion, culture, and PCR), the prevalence of the disease in the UK (6.4–11%) [8] and in Ireland (9–11%) [9] has recently decreased due to the control activities.

The prevalence of tuberculosis is lower in population badgers in France (4.57–5.14%) [10] and in Spain (4.23%) [11] than in Northern European countries. In France, the density of adult badgers was recently estimated to be between 1.66 and 7.86/Km^2^, with an average of 3.84 and a social group size between 1.34 to 4.46 [12]. Studies carried out in the Iberian Peninsula seemed to indicate a social strategy based on well-defined social units composed of 2–4 adults and a density of 0.23–0.67 badgers/Km^2^ in Spain [13] and of 0.53–0.94 badgers/Km^2^ in Portugal [14], less dense than that found in Northern Europe.

Tuberculosis in the badger is a respiratory disease. In a review, Gallagher and Clifton-Hadley (2000) [15] described in detail the gross pathology and the pathogenesis of tuberculosis in badgers. They reported various investigations carried out in Britain and Ireland in which infected badgers showed mainly lung lesions and broncho mediastinal node lesions. However, in contrast with cattle, in which gross lymph node lesions accompany lung lesions in the great majority of cases, in the badger, this occurred in a minority of cases. The highest percentage of lesion frequencies was bronchial, retropharyngeal, mediastinal, prescapular, and popliteal [15].

Lung lesions of tuberculosis in badger may vary from nodules of 1–3 mm to widespread lobar disease where miliary tubercles coalesce to form large masses. Pleurisy, either nodular or unilateral pyothorax, nodular epicarditis, and pericarditis were found on occasion [15].

Other Mycobacteria were isolated from badgers. A total of 5 (7.35%) and 281 (0.51%) isolates of *M. avium* spp. were recovered from badgers during previous studies in Spain and in the UK, respectively, but none of the infected badgers showed lesions containing a large number of MAC bacilli, so the authors concluded that they may not have a significant role in the transmission of MAC organism [16].

In Italy, the ecology of *Meles meles* is different from that of Northern Europe, and livestock is managed under semi-extensive regimes. From the Italian national database of the Livestock Registry [17], it appears that, in 2021, in the Abruzzo and Molise regions, 3.80% and 1.46% of cattle, respectively, moved to pastures, while greater movements to pastures were recorded in Piedmont (34.76%) and Trentino alto Adige (10.71%) [17].

In Italy, badgers dig dens or use those dug by foxes and porcupines, and differently from what happens in Northern Europe, they exhibit a solitary behavior, not forming social groups sharing the same den [18].

One possible explanation for this great difference is that the mild and damp climate of the British Isles, with little snow cover and few dry periods, together with the high prevalence of pasture, provides ideal conditions for Earthworms *Lumbricus terrestris*, which are their main prey. In continental Europe, including Italy, badgers have been found to specialize in very different foods, such as insects and fruits [19].

There are no studies on the density of badgers in the Abruzzo and Molise regions. Recent data document that in Northern Italy, social groups are formed by two to four adults, similar to what was described in Spain and Portugal. The badger density is 0.93–1.4/Km^2^ [20], which is rather high compared with the data of the Iberian Peninsula [13,14].

The badger is distributed throughout the Italian peninsula, while it is absent in Sicily, Sardinia, and smaller islands. The habitats of the badger are forests both in the plains and in the mountains, up to 2000 m above sea level; it prefers deciduous or mixed woods, alternating with open, bushy, stony, and uncultivated areas [18]. The ability to live in different environments and to adapt its diet to very varied ecological conditions makes this species widespread and relatively common throughout Italy, from the Alpine and Apennine mountainous areas to the agricultural ones, where the extent of natural vegetation is limited, in the dense scrub environments as well as in coastal areas [18].

The Abruzzo and Molise regions are totally included in the distribution range of the badger in Italy. The Abruzzo region covers 10,800 Km^2^, and the Molise region 4438 km^2^ with an elevation ranging from the sea level, about 2900 m high for the former and about 2200 m for the latter.

In these regions, the variations in the vegetation cover are expressed along an altitudinal gradient corresponding to the climate gradient. The territory is mainly mountainous and hilly, and the plain consists of a narrow coastal strip. There are several ecosystems: the coastal plains include mainly urbanized and cultivated environments with fragmented natural habitats. Mixed forests (turkey oak, downy, and beech tree) are located throughout the sub-montane areas, while beech forests and montane shrublands and grasslands occur towards the higher elevations [21,22].

*Meles meles* is a species protected by European [23] and Italian laws [24]. However, although this species does not have particular conservation problems, the collection of samples for research purposes is only possible in the event of traffic accidents or natural death.

The aim of this study was to show the results of the analyses carried out to detect the presence of *Mycobacterium* spp., *M. tuberculosis* complex, *M. avium* subsp. *avium* e *M. avium* subsp. *paratuberculosis* in the European badger population residing in the Abruzzo and Molise regions.

## 2. Materials and Methods

### 2.1. Study Area and Sample Collection

Between 2013 and 2021, the carcasses of 124 badgers, including 113 adults and 11 juveniles (63 females and 61 males), mainly involved in road accidents in Abruzzo and Molise regions, were examined at IZS-Teramo. The carcasses were found in the following provinces: 65 in L’Aquila, 32 in Chieti, 15 in Teramo, 4 in Isernia, 5 in Pescara, and 3 in Campobasso.

**Figure 1:**
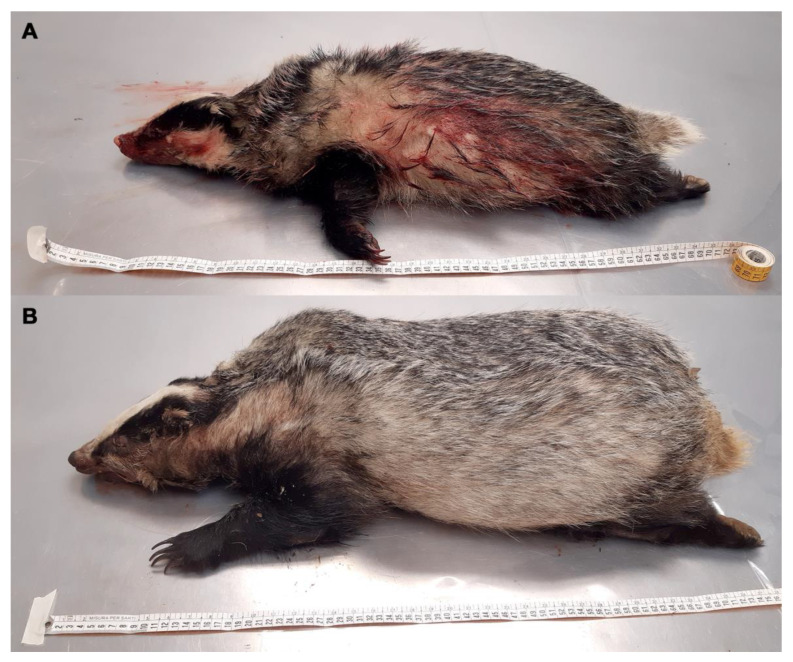
(**A**) Young badger carcass with tip nose-base tail length < 61 cm; (**B**) adult badger carcass with tip nose-base tail length > 61 cm.

A wide variety of methods were used to age badgers. These included techniques based on the fusion of tibial epiphyses, teeth sectioning, teeth wear, eye lens mass, and skull structure, but accurate aging of adult badgers remains problematic [25]. So, in this study, the classification into young and adult animals was carried out by measuring the distance from the tip of the nose to the base of the tail. Usually, in adult badgers, this distance is 61–73 cm (cm) [26]. Badgers with a length of less than 61 cm were considered young (Figure 1A,B).

### 2.2. Post-Mortem and Histological Examination

Each carcass was subjected to an anatomopathological examination. Head lymph nodes were collected from all carcasses, and mediastinal lymph nodes from 20 of them were used for bacteriological and molecular tests in the search for *Mycobacterium* spp.

The bacteriological analysis was performed on a small number of tissues for each carcass; differently from the more sensitive approach described by Corner et al. (2012) [6], our diagnostic strategy is used to obtain initial information in undisturbed wild population at not an excessive cost to gain insights into epidemiological patterns in field studies, as supported by Delahay (2012) [27]. As relative indices of infection, we can use them to identify long-term trends in space and time in naturally infected populations.

Samples of lung, spleen, liver, and mesenteric lymph nodes were also collected from 31 carcasses and were fixed in 10% neutral buffered formalin, embedded in paraffin, and routinely processed for histology (hematoxylin and eosin staining—H&E).

### 2.3. Bacteriology

The lymph node tissue samples were individually homogenized, decontaminated with 5% sulfuric acid, and subjected to the culture test for the isolation of *Mycobacterium* spp. [28]; each homogenate was inoculated into the Lowenstein–Jensen (LJ) solid medium [28]; the homogenate was also inoculated in the liquid medium of the Bactec 9120 MB system, according to the method reported in the manufacturer’s manual.

Growth on solid medium was monitored every week with visual examination. Instead, Bactec system warned the operator of the presence of positive samples by turning on a warning light. The suspicious colonies, grown during the eight weeks of incubation, were picked up with a needle, placed on a glass slide, and subjected to staining using the Ziehl Neelsen procedure (ZN). Colonies of acid-fast bacteria, fuchsia-colored, were subjected to molecular identification by DNA amplification. The absence of growth of characteristic colonies was interpreted as negative.

### 2.4. DNA Extraction and IS6110 PCR

The search for *Mycobacterium tuberculosis* complex by direct tissue PCR was performed from the homogenized and decontaminated tissue samples used for culture isolation.

A 200 µL aliquot of homogenized Lymph node sample and tissue was submitted to mechanical lysis using 100 µg of glass beads (100 to 200 µm in diameter) in a Qiagen tissue lyser apparatus for 5 min at 30 Hz. Subsequently, the purification and extraction of the DNA was carried out by using Maxwell^®^ 16 Tissue DNA Purification Kits (Technical handbook) and amplified with the PCR Multiplex technique through the specific amplification of a segment of 209 bp of the IS6110 insertion element according to the technique described by Boniotti et al. (2014) [29].

### 2.5. Genotyping of Mycobacterium spp. Isolates

For the identification of mycobacteria, BACTEC by-cultures containing colonies referable to *Mycobacterium* spp. and the isolates strains of *Mycobacterium* spp. have undergone DNA amplification with the DNA polymerase chain reaction (PCR) method [28,30,31,32]. The primers shown in Table 1 have been used for DNA amplification.

### 2.6. Statistical Analysis

Apparent prevalences of mycobacteria in these badgers were calculated and compared. Data from the Laboratory Information Management System were imported in MS Access^®^ (Microsoft Access 2019, Redmond, Washington, DC, USA), which was used for cleaning and normalizing the dataset. The 95% credibility intervals (95% CI) with the indication of lower (l.c.l.) and upper (u.c.l.) credibility levels were calculated using a Bayesian approach with a beta distribution (n + 1; n − s + 1), where n is the total number of tested samples, and s is the tested positive samples [33].

## 3. Results

**Figure 2:**
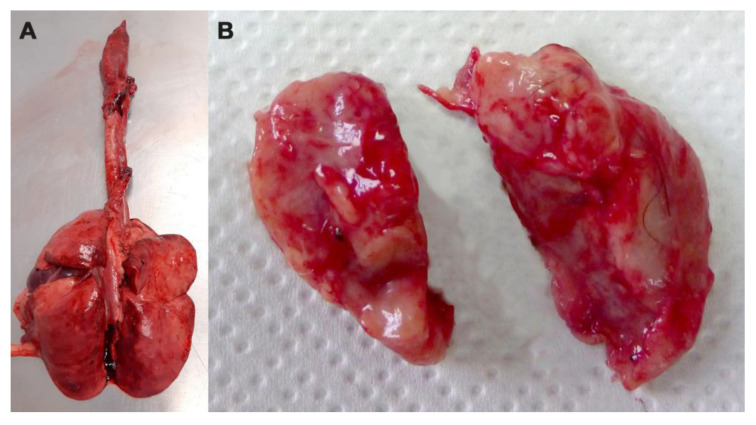
(**A**) Lungs and (**B**) retropharyngeal lymph nodes of road-killed badger without lesion of miliary tuberculosis.

The anatomopathological and histological analyses did not reveal the presence of tuberculous lesions in the organs of the examined animals (Figure 2).

In the 124 carcasses examined, mycobacteria were isolated from four animals (3.22%) (CI 95% 1.3–8.0), *M. avium* subsp. *avium* was isolated by head lymph nodes from two badgers (1.61%) (CI 95% 0.5–5.7), *M. avium* subsp. *paratuberculosis* (0.80%) (CI 95% 0.2–4.4) from one, and *Mycobacterium* spp. from another (0.80%) (CI 95% 0.2–4.4).

**Figure 3:**
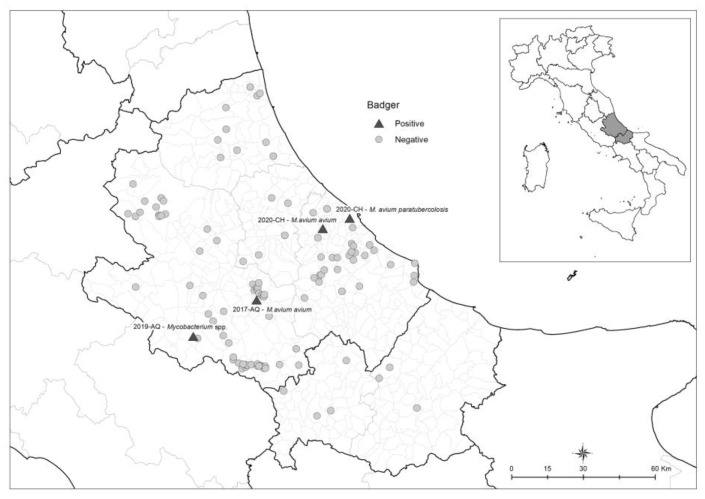
Map of tested and positive badgers in Abruzzo and Molise regions.

The largest number of carcasses was collected along the roads of the province of L’Aquila, near the municipalities of Sulmona and Alfedena, and in the province of Chieti, near the municipality of Guardiagrele and Lanciano, on secondary roads with little traffic (Figure 3). This is consistent with a study conducted in the Abruzzo region by Fabrizio et al. (2019) [34], which observed that the highest roadkill risk for badgers is characterized by intermediate values of both habitat suitability and landscape connectivity on isolated roads with little traffic rather than on large ones.

The search for *M. tuberculosis* complex from tissue homogenate with the PCR technique has resulted in negative for all the carcasses. The badger’s information is reported in Table 2.

**Figure 4:**
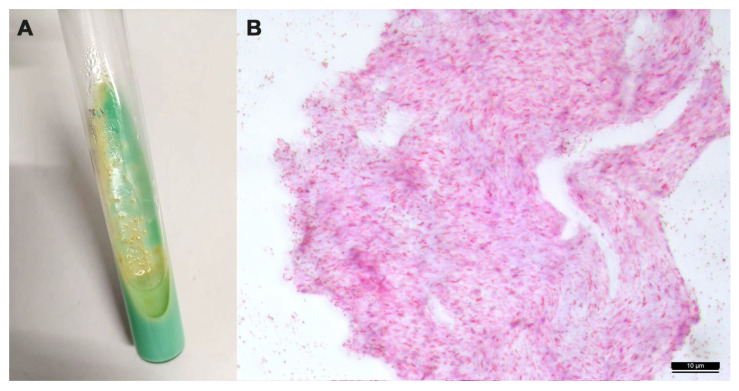
(**A**) Colonies of *Mycobacterium avium* subsp. *avium* grown on Lowenstein–Jensen medium; (**B**) Zielh–Neelsen positive staining of a suspected colony. Microscopic examination of the acid-fast smear reveals cells that are typically short and coccobacillary (100×).

In the LJ medium, the colonies of the isolate mycobacteria were slow-growing ones (>2 weeks to form visible colonies), initially very small, about 1 mm in diameter, colorless, translucent, and convex with a smooth and shiny surface; after prolonged incubation, the colonies showed an opaque, wrinkled, and raised appearance (Figure 4A). They were ZN-positive staining (Figure 4B).

## 4. Discussion

This study provides evidence of infection with the *M. avium* subsp. *avium* (MAA) and *M. avium* subsp. *paratuberculosis* (*MAP*) in badgers for the first time in Italy.

Infection with the MAC organism causes the formation of purulent, granulomatous, and caseous lesions [35], but no grossly visible lesions and no microscopic granuloma were observed in the four positive badgers identified in this Italian study. The lack of gross lesions in the tissues of the infected animals may be related to the phase of infection or to the potential resistance to MAA and MAP of this species, as described for *M. bovis*. In all reports on necropsy examinations of badgers, a significant percentage of cases are counted in which there is *M. bovis* in the absence of macroscopic lesions (no visible lesions NVL) [15]. In these reports, from histological findings, *Meles meles* shows a containment phase early in the pathogenesis of the disease, which results in a lengthy period of latency, even including years [15]. Latent cases may never progress further, but in some badgers, this state may be triggered by stressors to a serious disease [15]. In a survey carried out in Spain, the five *M. avium*-positive badgers did not present macroscopic lesions, but on histological examinations of the retropharyngeal and sub-mandibular lymph nodes, they showed small granulomas, positive for mycobacterial antigens [16]. Although acid-fast bacilli were not observed on the ZN staining of the tissue sections, positive immunolabelling for mycobacterial antigens was detected in both macrophages and lymphocytes within granulomas [16].

*M. avium* subsp. *paratuberculosis* causes a chronic intestinal infection that affects domestic and wild ruminants, but several species were described as susceptible to the disease and reservoirs for transmission between wildlife and domestic animals. Infection is usually acquired through the fecal–oral route, standing the possibility of inter-species transmission through the ingestion of grass contaminated by infected feces from domestic and wildlife animals, especially deer and rabbits [36].

In livestock, MAP can cause serious biological and economic damage. MAP infection has been detected on farms worldwide [37]. Prevalence studies report the presence of MAP in the regions bordering Abruzzo and Molise in ovine farms in the Marche region [38] in sheep and goat farms in Apulia [39] and cow farms in Umbria and five regions of Southern Italy [40,41]. In Italian wildlife, *M. avium* subsp. *paratuberculosis* has been detected in many wild ruminants [42], and in wild boars (*Sus scrofa*) in Sardinia [43]. Paratuberculosis was reported in Europe mainly in cervids. However, the transmission of MAP between rabbits and cattle has been reported, and a high prevalence of paratuberculosis in the wild rabbit population may be associated with a high prevalence in domestic animals [42]. MAP reservoirs may exist locally, but their significance for paratuberculosis control in livestock is quite limited [36].

The most critical aspect derived from MAP and MAA infections in wildlife is the possible interference with tuberculosis diagnosis (skin test); this is thought to be due to the shared antigens between NTM and MTBC [36,44,45,46,47]. In Northern Italy, a study suggested a possible inter-species transmission of the same genotype of *M. avium* subsp. *paratuberculosis* among wild red deer (*Cervus elaphus*) and farm cattle, but a whole genome sequencing of isolates is necessary to confirm the data obtained from VNTR typing [48]. The results suggest that all isolates share an identical VNTR profile corresponding to the INMV1 genotype. The only variation was on the locus SSR2, but the utility of this last locus has already been questioned because of its instability [48].

Differently from what has been reported in the United Kingdom, Ireland, France, and Spain, the results obtained in this study suggest that in Central Italy, *M. bovis* does not seem to circulate within the badger population.

In the Abruzzo and Molise regions, *M. bovis* was occasionally found in wildlife. In March 2014, *M. bovis* was isolated from multiple organs of a free-ranging Marsican brown bear (*Ursus Arctos Marsicanus*) and deceased for systemic tuberculosis in the province of L’Aquila, the territory of Abruzzo, Lazio, and Molise National Park. The animal was found alive and showed severe, non-specific clinical signs and died soon after its discovery by park rangers. A presumptive diagnosis of tuberculosis was initially made from gross findings, especially on the enlarged necrotic mesenteric lymph nodes and rectal necrotic plaques. Histopathology confirmed the tubercular nature of the infection, showing specific granulomatous lesions with the presence of acid-fast bacilli. By molecular characterization, this isolate resulted in identical to isolates from an outbreak in semi-free-ranging bovine animals, which had been first detected in June 2012 in the Gioia dei Marsi municipality (AQ) [49]. It was ascertained that *M. bovis*-infected cattle had been grazing in the home range of this bear since 2012. Some infected bovines died on pastures and were consumed by scavengers [49]. In 2023, *M. bovis* was isolated from the organs of a free-ranging red deer (*Cervus elaphus*) in the same municipality, and its molecular characterization is ongoing [Tieri, personal communication]. In the 2019–2022 period, MTBC bacteria were observed in four wild boars (three from Abruzzo and one from Molise regions), and the molecular identifications are ongoing [Tieri, personal communication].

To maintain the disease-free status from MTBC infection in these territories, a stricter application of health regulations in force is warranted to control the presence of bovine tuberculosis in areas where wildlife and cattle share the same pastures. Trades and the practice of summer translocation to mountain pastures should be fully considered among the risk factors for the introduction of bovine tuberculosis in protected areas [49].

Currently, in Italy, bovine tuberculosis prevalence is variable: regions located in the north and center of the Italian peninsula and in Sardinia are officially recognized as free, including, recently, Abruzzo and Molise (Regulation EU 2021/620 and subsequent amendments) [50], while the highest prevalence is recorded in the south and, above all, in Sicily (5.21%) [51]. In this insular region, where the badger is not present, studies have suggested that the Sicilian black pig (*Sus scrofa*) acts as a reservoir of bovine tuberculosis in the Nebrodi ecosystem (Messina province) [52,53]. The detection of *M. bovis* DNA in swine feces confirmed the active excretion of the pathogen through feces, highlighting the swine’s role in environmental contamination [53].

## 5. Conclusions

Although this study found evidence of MAA and MAP bacteria in badgers, none of the infected animals showed lesions containing a large number of MAC bacilli, so *Meles meles* should not play a role in the epidemiology of MAC in the territory of the Abruzzo and Molise regions under current epidemiological conditions.

However, the detection of MAC in three badgers tested in this study suggests the possibility that this species, in the territories likely to be shared with cattle, could be a vehicle of these bacteria and, therefore, could potentially interfere with the surveillance activities carried out in the context of the National Eradication plan for tuberculosis in cattle. This leads to the possible consideration of the need for a systematical comparative antemortem screening test (skin test or IFN-y test) in the final steps of the eradication program.

## Figures and Tables

**Table 1:** List of the specific primers used for DNA amplification with PCR method.

*Mycobacterium* Target	Name of the Primer	Primer Sequencing	Target Gene	Amplicon Size
*Mycobacterium* spp.	Mycgen-F	5′-AGAGTTTGATCCTGGCTCAG-3′	mbp70	1030 bp
	Mycgen-R	5′-TGCACACAGGCCACAAGGGA-3′		
*M. tuberculosis* complex	TB1-F	5′-GAACAATCCGGAGTTGACAA-3′	mbp70	372 bp
	TB1-R	5′-AGCACGCTGTCAATCATGTA-3′		
*M. avium paratuberculosis*	DMC1	5′-GATCGGAACGTCGGCTGGTCAGG-3′	IS900	217 bp
	DMC2	5′-GATCGCCTTGCTCATCGCTGCCG-3′		
*M. avium avium*	Mycgen-F	5′-AGAGTTTGATCCTGGCTCAG-3′	mbp70	180 bp
	Mycav-R	5′-ACCAGAAGACATGCGTCTTG-3′		

**Table 2:** Characteristics of mycobacteria positive badgers.

General Register Number	Date of Discovery	Municipality of Discovery	Sex	Age	Cause of Death	Tissues Examined	Methods	*Mycobacterium* Species
4860AZ2017	11 September 2017	Introdacqua (AQ)	F	Adult	Road-killed	Head lymph nodes	Culture+; BACTEC- PCR-	*M. avium* subsp. *avium*
4804AZ2019	4 September 2019	Collelongo (AQ)	F	Adult	Road-killed	Head lymph nodes	Culture+; BACTEC+ PCR-	*M.* spp.
41362TE2020	7 May 2020	Ortona (CH)	M	Adult	Predation	Head lymph nodes	Culture+; BACTEC+ PCR-	*M. avium* subsp. *paratuberculosis*
298930TE2020	26 November 2020	Vacri (CH)	M	Young	Road-killed	Head lymph nodes	Culture+; BACTEC+ PCR-	*M. avium* subsp. *avium*
						Mediastinal lymph nodes	Culture-; BACTEC-; PCR-	
						Lung	Histological exam: absence of tubercular lesion	

## Data Availability

The data used in this study are the property of Istituto Zooprofilattico Sperimentale dell’Abruzzo e del Molise “G. Caporale”, Italy, and thus not publicly available.
